# Contrast Enhancement in Breast Cancer: Magnetic Resonance vs. Mammography: A 10-Year Systematic Review

**DOI:** 10.3390/diagnostics14212400

**Published:** 2024-10-28

**Authors:** Francesco Filippone, Zohra Boudagga, Francesca Frattini, Gaetano Federico Fortuna, Davide Razzini, Anna Tambasco, Veronica Menardi, Alessandro Balbiano di Colcavagno, Serena Carriero, Anna Clelia Lucia Gambaro, Alessandro Carriero

**Affiliations:** 1 SCDU Radiology, “Maggiore della Carità” Hospital, University of Eastern Piedmont, 28100 Novara, Italy; franzi.frattini@gmail.com (F.F.); gaetanofederico.f@gmail.com (G.F.F.); davide_razzini@tiscali.it (D.R.); annatambasco11@gmail.com (A.T.); menardiveronica@gmail.com (V.M.); alessandrobalbianodc@gmail.com (A.B.d.C.); gambaroa@yahoo.it (A.C.L.G.); profcarriero@virgilio.it (A.C.); 2 Foundation IRCCS Cà Granda-Ospedale Maggiore Policlinico, 20122 Milan, Italy; serena.carriero@unimi.it

**Keywords:** breast cancer, contrast media, MRI, CEMR, CEM, allergic reaction

## Abstract

PURPOSE: Contrast Enhancement Magnetic Resonance (CEMR) and Contrast-Enhanced Mammography (CEM) are important diagnostic tools to evaluate breast cancer patients, and both are objects of interest in the literature. The purpose of this systematic review was to select publications from the last ten years in order to evaluate the literature contributions related to the frequency of contrast agents used, administration techniques and the presence of adverse reactions. METHODS: We have selected, according to the PRISMA statement, publications reviewed on Pub Med in the period from 1 January 2012 to 31 December 2022. The search engine was activated using the following keywords: “CESM”, “CEM”, “CEDM”, “Contrast mammography” for CEM, “DCE-MRI”, “Contrast Enhancement MRI” for CEMR, excluding reviews, book chapters and meta-analyses. From the total number of publications, we made a preliminary selection based on titles and abstracts and excluded all articles published in languages other than English and all experimental studies performed on surgical specimen or animal population, as well as all articles for which the extended version was not available. Two readers evaluated all the articles and compiled a pre-compiled form accordingly. RESULTS: After a preliminary collection of 571 CEM publications, 118 articles were selected, relating to an overall population of 21,178 patients. From a total of 3063 CEMR publications, 356 articles relating to an overall population of 45,649 patients were selected. The most used contrast agents are Iohexol for CEM (39.83%) and Gadopentetic acid (Gd-DTPA) for CEMR (32.5%). Regarding the CEM contrast administration protocol, in 84.7% of cases a dose of 1.5 mL/kg was used with an infusion rate of 2–3 mL/s. Regarding the CEMR infusion protocol, in 71% of cases a dose of 1 mmol/kg was used at an infusion rate of 2–4 mL/s. Twelve out of 118 CEM articles reported allergic reactions, involving 29 patients (0.13%). In DCE-MRI, only one out of 356 articles reported allergic reactions, involving two patients (0.004%). No severe reactions were observed in either cohort of exams. CONCLUSIONS: CEM and CEMR are essential contrast methods to evaluate breast diseases. However, from the literature analysis, although there are preferences on the uses of the contrast agent (Iohexol for CESM, G-DTPA for CEMR), a wide range of molecules are still used in contrast methods, with different administration protocols. Based on the collected data, it is possible to state that both methods are safe, and no severe reactions were observed in our evaluation.

## 1. Introduction

Breast cancer is one of the most common neoplastic pathologies in the female population [1,2,3] and is a subject of particular interest in the scientific literature.

The definition of breast cancer actually refers to a group of diseases that vary in histology, methods of presentation, aggressiveness, clinical behavior [4,5] and prognostic factors [6,7,8,9]. The constant technological evolution of various imaging techniques has made a notable contribution to the early diagnosis and management of breast cancer [10,11,12]. However, it is with contrast methods, such as Contrast Enhancement Magnetic Resonance (CEMR) [13,14,15,16,17] and Contrast Enhancement Mammography (CEM) [18,19,20], that breast cancer imaging has been revolutionized [21,22]. So, contrast media is actually a unique tool in breast imaging, even if the most recent researches about IA in breast field are very promising for the extension of the data extracted from non-enhanced mammography and MRI [23].

The importance of contrast imaging and the resulting high diagnostic accuracy has its rationale in the phenomenon of tumor neoangiogenesis, i.e., in the intrinsic capacity that a tumor has to create new blood vessels to guarantee the supply of oxygen and glucose necessary for its existence, producing dysmorphic and fenestrated capillaries responsible for the peculiar contrastographic behavior of heteroplastic lesions [24,25,26,27].

It is well-established that tumor growth and metastasis development are angiogenesis-dependent processes [28]. These newly formed vessels are disorganized and irregular, and the basement membrane is either discontinuous or altogether absent, further increasing the permeability of tumor vessels.

The angiogenesis process plays a central role not only in the comprehension of breast cancer pathogenesis [29] but also in the development of novel therapeutic strategies, such as monoclonal antibodies targeting the vascular endothelial growth factor (VEGF) [30] and immune checkpoint inhibitor (ICI) drugs [31]. In fact, given the central role that VEGF receptor 2 plays in neoangiogenesis, it is the target of numerous anti-tumor therapies, and quantitative imaging of its expression is proving to be a new multiparametric approach to breast cancer [32].

In this scenario, contrast agents used in CEM and CEMR exploit the increased permeability of abnormal tumor vessels to highlight breast lesions [33,34].

So, these methods represent a crucial step in the diagnostic path of breast cancer.

The aim of our work was to analyze the publications of the last ten years relating to CEM and CEMR contrast methods in order to evaluate the frequency of the contrast agents used, the administration technique and the presence of adverse reactions.

## 2. Materials and Methods

This systematic review was reported according to the Preferred Reporting Items for Systematic Reviews and Meta-Analyses (PRISMA) statement [35].

The literature review was performed in electronic databases PubMed for both CEM and CEMR studies and covered the period between January 2012 and December 2022.

To select the CEM studies, the following keywords were used: “CESM”, “CEM”, “CEDM” and “contrast enhanced mammography”. To select the CEMR studies, the keywords used were: “BREAST DCE-MRI” and “Breast Contrast Enhancement MRI”. Books and Documents, Meta-Analysis, Review and Systematic Review were excluded using the search engine tool.

A preliminary list of articles was compiled, and subsequently all collected articles were cross-checked to identify and eliminate duplicates. Articles which evaluate both CEM and CEMR were considered as two different articles and assessed separately.

Two fourth-year radiology residents with breast cancer experience were tasked with the preliminary selection of articles of interest based on the abstracts and titles.

Subsequently, the following exclusionary criteria were applied to the selection made:

- Articles written in any language other than English were excluded.
- Experimental studies performed on surgical specimen, animal population, modeling.
- Studies not involving a living, quantifiable human population.
- Studies where the full text versions were unavailable.

**Figure 1:**
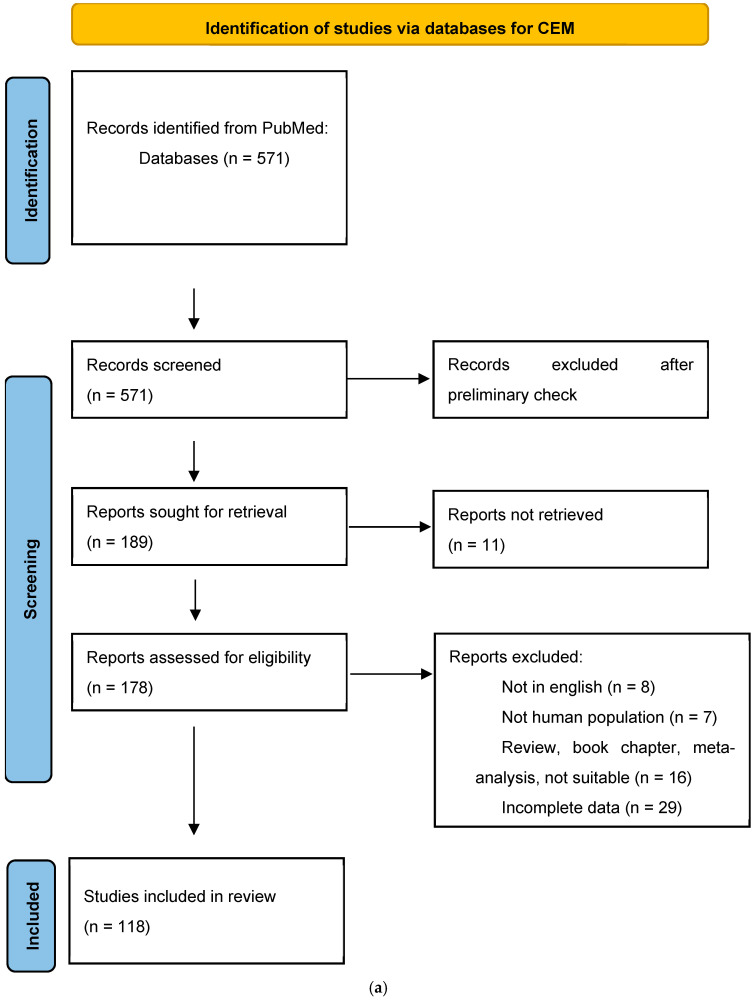

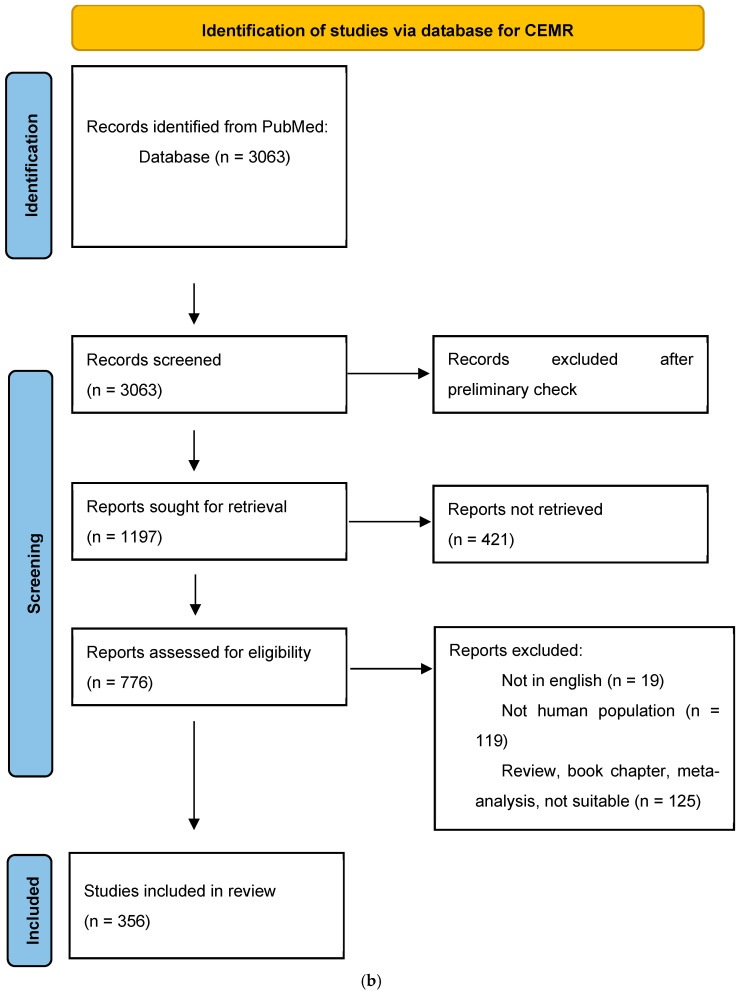
(**a**) Flowchart showing the study selection process for CEM; (**b**) Flowchart showing the study selection process for CEMR.

Details are summarized in Figure 1a,b.

Subsequently, two radiologists with over 5 years of experience of breast cancer evaluated the articles and for each article compiled a form which included the evaluation of CEM and CEMR cases according to the following information:

- Population number.
- Contrast agent molecule.
- Contrast agent administration technique, quantity per kilo, flow rate (mL/s).
- Adverse reaction to contrast agent.

When reactions to the contrast agent are indicated, both the number of allergic reactions and the severity were noted.

## 3. Results

### 3.1. CEM

From the total number of articles initially chosen via the PubMed search engine (571), a selection was carried out, in compliance with all the exclusion criteria reported, resulting in a selection of 118 articles published from January 2012 to December 2022.

Five articles were case reports, each involving a single patient. Excluding case reports, the populations studied ranged from a minimum of 11 patients [36] to a maximum of 1239 [37], for a total number of 21,178 patients.

**Figure 2:**
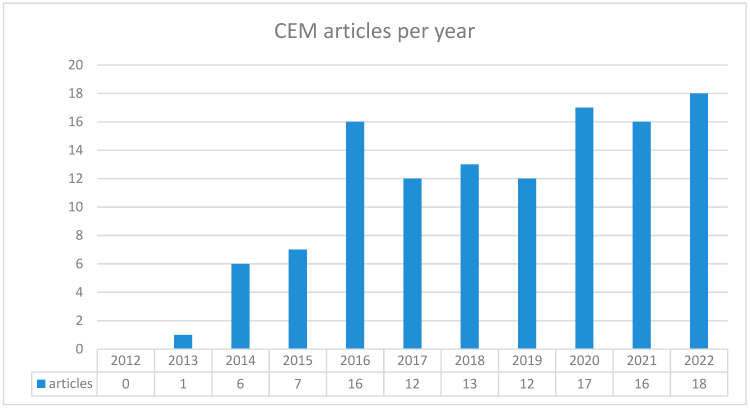
Graphic showing the number of CEM articles evaluated sorted per year.

Details on the number of articles per year are expressed in Figure 2.

### 3.2. Contrast Agent Data for CEM

Out of 118 articles examined, 15 (12.7%) referred to 3902 patients (18.4%) and generally indicated that an iodinated contrast medium was administered without specifying the molecule. In contrast, 11 articles (9.3%), referring to 1280 patients (6%), did not mention the contrast medium.

In the remaining 92 articles (78%) relating to 16,066 patients (75%), information relating to the contrast agent molecule was provided.

Seven different types of contrast agent were used (Iohexol, Iodixanol, Iopromide, Iomeprol, Ioversol, Iobitridol and Iopimadol). The most used contrast agent was based on the molecule Iohexol, administered in 47 out of 118 studies (39.8%) and to 7109 out of 21,178 patients (33.5%), followed by Iopromide, administered in 22 studies (18.6%) and 3849 patients (18.17%). Details are shown in Table 1.

Although 16 studies poorly reported the details of the contrast agent administration, in 100 of 118 studies (84.7%), the contrast agent was administered in quantities of 1.5 mL/kg at a flow rate between 2 and 4 mL/s.

### 3.3. Adverse Reactions Data for CEM

Out of 118 articles, 51 (43%) do not mention adverse reactions to the contrast medium, and in 55 studies (46%) it is reported that the study population was selected by excluding patients with a history of adverse reactions to the contrast medium. Finally, out of 118 studies, adverse reactions were observed in 12 (10%) publications and involved 29 patients out of 21,178 (0.13%).

Details are shown in Table 2.

Of 29 patients who experienced adverse reactions, 21 suffered only mild skin reactions (rash, urticaria, itching). Medium-grade reactions were observed in eight cases, with nausea, vomiting and skin reactions as reported symptoms. In both cases, symptoms regressed rapidly after administration of corticosteroids. No severe reactions were observed.

Of the twelve studies where reactions to the contrast medium were reported, Iohexol and Iopromide were responsible for allergic reactions in ten and seven patients, respectively, divided into four studies for the Iohexol and three studies for Iopromide. Considering the entire population of patients who were administered Iohexol, only 0.14% (ten out of 7109 patients) suffered allergic reactions, while the most allergenic contrast agents were found to be Iodixanol (two out of 762 patients, 0, 26%) and Iopromide ( out of 3849 patients, 0.18%). Details are provided in Table 3.

### 3.4. CEMR

From a total of 3063 articles selected with the search engine, after a selection in compliance with all the exclusion criteria indicated, 356 articles were selected, published between January 2012 and December 2022.

The study populations varied from a minimum of four patients [50] to a maximum of 1979 [51], for a total number of 45,649 patients.

**Figure 3:**
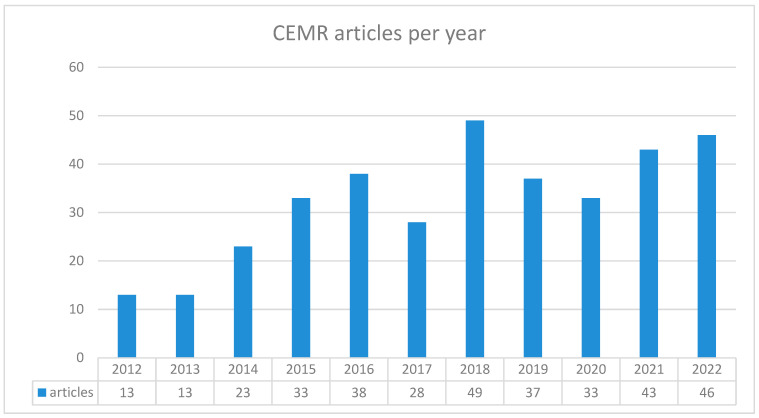
Graphic showing the number of CEMR articles evaluated sorted per year.

Details on the number of articles per year are expressed in Figure 3.

### 3.5. Contrast Agent Data for CEMR

Out of 356 publications, 27 (7.58%) referred to 2009 patients (4.4%) and generally indicated that a gadolinium-based contrast agent was administered, while in 33 studies (9.2%) referring to 6317 patients (13.8%) the contrast agent was not mentioned.

In the remaining 296 articles (83%) relating to a total population of 37,323 patients (81.7%), information relating to the contrast agent molecule was provided.

Six different gadolinium-based contrast agents were used (Gadoterate meglumine, Gadodiamide, Gadopentetic acid, Gadobutrol, Gadobenate dimeglumine and Gadoteridol). The most commonly used contrast agent was Gadopentetic acid (Gd-DTPA), administered in 116 studies (32.58%) to 16,303 patients (35.7%), followed by Gadobutrol, administered in 68 studies (19.1%) to 6580 patients (14.4%), and Gadiodiamide, administered in 38 studies (10.64%) to 6550 patients (14.3%). Details are shown in Table 4.

Out of 356 publications, in 253 (71%) the contrast agent was administered in quantities of 0.1 mmol/kg, at a flow rate between 2 and 4 mL/s. In 57 studies (16%), the contrast agent was administered in quantities between 0.05 to 0.5 mmol/kg. In 44 studies (12%), the details of the contrast agent administration were poorly reported.

### 3.6. Adverse Reactions Data for CEMR

Out of 356 articles, 340 (95%) do not mention adverse reactions to the contrast agent, and in 15 (4%), it is reported that the study population was selected by excluding patients with a history of adverse reaction to the contrast medium. Finally, out of 356 publications, adverse reactions were observed in one article (0.3%), involving two patients out of 185 of the study population (1%) and 45,649 patients of the total population (0.003%).

Both patients presented headache and nausea as symptoms. The contrast agent in this single study was Gadobutrol [46]. The data are reported in Table 2 and Table 3.

## 4. Discussion

Regarding the CEM publications, a total of 21,178 patients were analyzed and divided into 118 articles. Without considering the five case reports analyzed, each concerning a single patient, the article with the narrowest population, published by Barra et al., had a population of only 11 patients and was dedicated to evaluating CEM as an instrument for estimating residual tumor size after neoadjuvant chemotherapy [36]. The publication with the largest population, 1239 patients, is a retrospective multicenter study on attention-based deep learning in breast cancer research in CEM [37].

The different iodinated contrast agents are listed in Table 1. The contrast agent most used in CEM studies is based on the Iohexol molecule, which represents not only the most used molecule in study protocols (47 of 118, 39.83%) but also the molecule administered to the largest proportion of patients included in this review (7109 of 21,178, 33.57%). Iohexol is a non-ionic, low-osmolarity contrast agent which, to date, represents the most chosen contrast agent in many radiology departments. Over the years, several dedicated studies seem to indicate that Iohexol has a greater tolerability and a lower tendency to give side effects [52], even in patients with a history of adverse reactions to iodinated contrast [53]. Wang CL et al. also analyzed the long-term consequences of patients with reactions to Iohexol, reporting little tendency for the molecule to cause severe reactions or long-term sequelae [54].

There was also a general consensus in the method of administration of contrast agent at a dose of 1.5 mL/kg and with a flow of 2–3 mL/s, as observed in 100 studies (84.7%). The data are substantially similar to what was observed by Zanardo et al. in 2019; however, concern was raised that these parameters had been adopted empirically via the CT method, and this aspect is rarely explored in depth in the various studies [55,56].

A potentially interesting fact to observe is that in 15 studies (12.7%) concerning 3902 patients (18.4%), the use of an iodinated contrast agent was generally indicated, while in 11 (9.3%) studies with a total population of 1280 (6%) patients, the contrast agent was not mentioned, not even in the specific section. It is assumed that the molecule used was not mentioned because it is unknown, as these are retrospective studies. Other hypotheses are that different molecules were used, or that the data about contrast media administration was considered pleonastic or unnecessary as it was not the purpose of these studies.

Regarding adverse reactions to the contrast agent, out of 118 articles examined, in 51 (43%), no data on the adverse reaction is mentioned, while in 55 (46%) studies it is specified how patients with a history of allergic reactions to the contrast agent have been previously excluded from the population, or it is directly specified that no allergic reactions have been observed. Finally, there are 12 (10%) studies where an adverse reaction to the organ-iodinated contrast agent is reported (Table 2); 29 patients out of the 21,178 examined (0.13% were affected by allergic reactions after administration of iodinated contrast agent.

In detail, 70% of allergic reactions observed were simply mild skin reactions that quickly regressed. Kim G et al. reports that a patient experienced an allergic reaction with facial rash, but that she presented symptoms 4 days after the procedure and therefore the correlation between the reaction and the administration of the contrast agent cannot be assured [41]. Houben IPL et al. reports, in addition to four minor skin reactions, a moderate reaction that required medical treatment, but without specifying the exact nature or symptoms [44].

Our data tend to confirm the very low quantity of reactions, already observed in the meta-analysis by Zanardo et al., which recorded 30 adverse reactions in 14,012 patients, and in the studies dedicated to the CT method by Wang et al. which identified reactions in 0.6% of 84,928 [54] and Mortelé et al., which reports 0.7% in 29,508 patients [57]. In our meta-analysis, no severe reactions were reported in the evaluated study; Wang et al. reported severe reactions in 11 out of 84,928 (0.0129%) and Mortelé et al. in four out of 29,508 (0.0135%). Zanardo et al. only report one severe reaction in 14,012 (0.007%) patients referenced from Dieckmann et al., but this study was published in 2011 and therefore was not included in our study cohort [58].

Evidently, as also reported in the aforementioned studies, these data must be considered with caution, given the rarity of adverse events associated with the contrast agent and the different clinical profiles of patients undergoing CT examinations, who are generally more severely affected compared to patients undergoing CEM.

Taking contrast agents into consideration, we see an almost ubiquitous distribution, with six of the seven molecules involved in allergic reactions. The molecules involved in multiple studies with allergic reactions are Iohexol and Iopromide, responsible for allergic reactions in ten and seven patients divided in four and three studies, respectively. If we consider the entire population to which Iohexol was administered, only 0.14% (10 out of 7109 patients) presented allergic reactions, a significantly lower number if compared with other molecules such as Iodixanol (two out of 762, 0.26%) and Iopromide (seven of 3849, 0.18%). Again, these data seem to confirm a lower tendency of Iohexol and in general of low-osmolarity contrast agents to engender allergic reactions in patients to whom it is administered [54,55,56].

As regards the CEMR articles, a total of 45,649 patients were analyzed, divided into 356 studies. The article with the smallest population is that of Zhang L et al., a pilot study aimed to evaluate MRI characteristics in papillary carcinoma in four patients [50]. Hu Q et al. is the publication with the largest population, 1971 patients, and represents a retrospective study on the study of benign and malignant breast lesions using deep features maximum [51].

The most used contrast agent in DCE-MRI studies is based on Gadopentetic acid (Gd-DTPA) used in 114 study protocols (41.9%) and administered to 15,849 patients (31%). Other widely used contrast agents are Gadodiamide (Gd-DTPA-BMA), used in 38 studies (10.6%) and administered to 6550 patients (14.3%), and Gadobutrol, used in 68 studies (18.49%) and administered to 6580 patients (14.4%). Gadopentetic acid (Gd-DTPA) is a molecule widely used in contrast-enhanced magnetic resonance imaging studies, whose efficacy as a contrast agent and safety has been well documented in several meta-analyses and trials published between 1987 and 2001. Regarding adverse reactions, the allergenic profile has proven to be particularly safe both locally and systemically, regardless of the route of administration [59]. As observed in CEM studies, there was a conspicuous number of articles that did not specify the contrast agent used, generally citing having used a gadolinium contrast agent in 27 studies (7.5%) concerning 2009 patients (4, 4%). However, there are 33 studies (9.27%) where no mention is made of the type of contrast used, concerning a total population of 6317 patients (13.8%). Our considerations in this regard are the same as those reported in the CEM section.

Compared to the CEM studies, there is less consensus in the contrast agent administration protocol. The most common protocol, observed in 253 publications (71%), involves the administration of a contrast agent at a concentration of 0.1 mmol kg, with a flow rate varying from 2 to 4 mL/s.

Compared to the CEM studies, the data regarding allergic reactions in the MRI studies were unremarkable; out of 356 studies examined, 340 (95%) do not mention adverse reactions to the contrast agent anywhere, neither in the body of the article nor in the abstract. Fifteen publications (4%) simply inform us that patients with a history of adverse reactions were already excluded from the final study population, and three of them specify that no adverse reactions were observed. Finally, out of 356 studies, adverse reactions were cited in only one publication (0.3%), which involved two patients out of a study population of 185 (1%), among 6580 who were administered the same molecule (0.03%), and out of 45,649 patients of the total population (0.003%). Both patients presented with headache and nausea, and the contrast agent in this single study was Gadobutrol [46].

This result could be justified by the fact that few allergic reactions to Gadolinium-based contrast agents are recognized in the recent literature. In a 2018 study, Davenport et al. reported how gadolinium-based contrasts have a low risk of causing allergic reactions, with incidence variability from 0.015% to 0.91%, which drops to 0.0016%/0.019% for severe grade reactions [60].

Similarly, Prince et al. reported not only that the incidence of allergic reactions is around 0.004%, but that 78.7% are mild and self-limiting allergic reactions, or which rapidly regress with contrast agents [61]. Kodwa R. et al. also report how severe reactions to gadolinium contrast agents are particularly rare [62]. The same data were also reported in Power et al., which also reports how Gadobutrol tends to give mild and quickly resolvable reactions [63].

Considering all this, we can look upon contrast media as innocuous. However, when discussing their safety profile from a wider point of view, we have to mention the rising role of IA and radiomics about breast cancer detection [23], and even molecular profile identification, using radiomic features extracted from mammography without contrast [64]. Recent studies, for example, have shown how integrating IA models into clinical practice could improve breast arterial calcifications (BAC) reporting without increasing clinical workload [65] and could distinguish dense from non-dense breast with accuracy of 89.3% [66].

## 5. Conclusions

Contrast Enhancement Magnetic Resonance (CEMR) and Contrast-Enhanced Mammography (CEM) are recognized as essential methods for evaluating breast cancer. Although there are preferences on the choice of molecule involved (Iohixol for CEM, Gabapentenic acid in CEMR), a wide range of molecules with different administration protocols are used. Based on the data collected, it is possible to state that both methods are safe for anaphylactic events. In fact, the reported allergic reactions did not have a real clinical impact, and no severe reactions were observed in our research. The large number of studies which do not devote sufficient space to the topic of allergic reactions is worrying. Despite this, the total number of reported allergic reactions is very low and in line with the data in the literature. Therefore, we can consider both CEMR and CEM methods as safe and the risk of allergic reactions negligible.

## Figures and Tables

**Table 1:** Table summarizing the CEM contrast agent molecule distribution based on the number of articles reporting its use and the number of patients to whom it was administered. The most commonly used molecule was Iohexol, administered in 47 articles (39.83%) to 7109 patients (33.57%).

CEM Contrast Agent Molecule				
Contrast Agent	Articles	% On Total	Patients	% On Total
Iohexol	47	39.83%	7109	33.57%
Iopromide	22	18.64%	3849	18.17%
Iopamidol	9	7.63%	3079	14.54%
Iodixanol	6	5.08%	762	3.60%
Iobitridol	4	3.39%	571	2.70%
Iomeprol	2	1.69%	518	2.45%
Ioversol	2	1.69%	108	0.51%
Iodine contrast agent	15	12.71%	3902	18.42%
Not specified	11	9.32%	1280	6.04%
	118		21,178	

**Table 2:** Table listing the 12 CEM articles and the single CEMR article where adverse reactions to the contrast medium was reported. For each article, among other data, the molecule used, the population number, the number of patients with adverse reaction and the nature of the reaction were reported.

CEM Allergic Reaction						
		Molecule	Dose	Flux	Pzn	Reaction
2021	Akmaral S et al. [38]	Iodine contrast agent	1.5 mL/kg	3 mL/s	184	5 skin reactions
2020	Sorin V et al. [39]	Iopamidol	1.5 mL/kg	3 mL/s	138	1 mild skin reaction
2020	La Forgia D et al. [40]	Iodixanol	1.5 mL/kg	3 mL/s	52	2 mild skin reactions
2019	Kim G et al. [41]	Iohexol	1.5 mL/kg	2 mL/s	64	1 mild skin reaction
2018	Sorin V et al. [42]	Iopamidol	1.5 mL/kg	3 mL/s	611	3 mild skin reactions
2018	Kim EY et al. [43]	Iohexol	1.5 mL/kg	2 mL/s	84	1 moderate nausea, vomit
2017	Houben IPL et al. [44]	Iopromide	1.5 mL/kg	3 mL/s	839	4 mild skin reaction, 1 moderate
2016	Tsigginou A et al. [45]	Iopromide	1.5 mL/kg	2–3 mL/s	216	1 mild skin reaction
2015	Chou CP et al. [46]	Iohexol	1.5 mL/kg	3 mL/s	185	6 moderate reaction with nausea, vomiting, skin flushing
2015	Hobbs MM et al. [47]	Iohexol	/	/	49	2 skin reactions
2014	Lobbes MB et al. [48]	Iopromide	1.5 mL/kg	3 mL/s	113	1 skin reaction
2014	Fallenberg EM et al. [49]	Iobitridol	1.5 mL/kg	3 mL/s	118	1 skin reaction
CEMR allergic reaction						
2015	Chou CP et al. [46]	Gadobutrol	0.1 mmol/kg	/	185	2 moderate headache, nausea

**Table 3:** Table listing the contrast agent molecules, sorting them by number of articles and patients in which they caused allergic reactions.

Adverse Reaction Compared to Contrast Agent Molecule				
	Articles	Patients	Per Total (%)	
CEM				
Iohexol	4	10	7109	0.14%
Iopamidol	2	4	3079	0.13%
Iodixanol	1	2	762	0.26%
Iopromide	3	7	3849	0.18%
Iobitridol	1	1	571	0.18%
Iodine contrast agent	1	5	3902	0.13%
CEMR				
Gadobutrol	1	2	6580	0.03%

**Table 4:** Table summarizing the CEMR contrast agent molecule distribution based on the number of articles reporting its use and the number of patients to whom it was administered. The most commonly used molecule was Gabapentenic acid (Gd-DTPA), administered in 116 articles (32.58%) to 16,303 patients (35.71%).

CEMR Contrast Agent Molecule				
Contrast Agent	Articles	% On Total	Patients	% On Total
Gabapentenic acid (Gd-DTPA)	116	32.58%	16,303	35.71%
Gadobutrol	68	19.10%	6580	14.41%
Gadoterate meglumine	38	10.67%	4179	9.15%
Gadodiamide (Gd-DTPA-BMA)	38	10.67%	6550	14.35%
Gadoteridol	19	5.34%	1866	4.09%
Gadobenate dimeglumine	17	4.78%	1845	4.04%
Gadolinium-based contrast agent	27	7.58%	2009	4.40%
Not specified	33	9.27%	6317	13.84%
	356		45,649	

## Data Availability

The data presented in this study are available on request from the corresponding author.
